# SNES makes sense? Single-cell exome sequencing evolves

**DOI:** 10.1186/s13059-015-0650-0

**Published:** 2015-04-29

**Authors:** Thierry Voet, Peter Van Loo

**Affiliations:** Department of Human Genetics, Laboratory of Reproductive Genomics, KU Leuven, 3000 Leuven, Belgium; Wellcome Trust Sanger Institute, Single-Cell Genomics Centre, Hinxton, CB10 1SA UK; The Francis Crick Institute, London, WC2A 3LY UK; Department of Human Genetics, Human Genome Laboratory, KU Leuven, 3000 Leuven, Belgium

## Abstract

Technologies for single-cell sequencing are improving steadily. A recent study describes a new method for interrogating all coding sequences of the human genome at single-cell resolution.

See related research by Leung *et al*., http://genomebiology.com/2015/16/1/55

## Introduction

In a recent report in *Genome Biology* [1], Nick Navin and colleagues develop a novel method for single-cell sequencing, single-nucleus exome sequencing (SNES). The method involves sorting single nuclei, a limited amplification step, exome capture and sequencing. The authors report good coverage across the exome and high detection efficiencies for finding single-nucleotide variants (SNVs) and small insertions and deletions (indels). The SNES method exemplifies the current progress and challenges in single-cell sequencing technologies [1].

Genomics studies have classically been performed on populations of cells, obscuring variability within cell populations. Mainly driven by the cancer field, significant advances have been made in computational methods that enable the characterization of different cell populations from the sequences of DNA extracted from a pool of many cells. However, accurate identification and characterization of low-abundant populations of cells requires bespoke experimental approaches. Over the past few years, several groups have pioneered the development of methods that allow profiling the DNA of single cells for diverse types of genetic variation (reviewed in [2]), and in fact in 2011 Navin *et al.* [3] published the first method for high-resolution copy number profiling using single-nucleus sequencing. A method that can read all 6 billion bases of a diploid human cell without errors and find all kinds of genetic variation is still to come, and will require major technical challenges to be overcome. The recent report in *Genome Biology* [1] illustrates how single-cell sequencing technologies are progressing in tackling those challenges. Such technologies may enable answering important questions in genetics, developmental biology and cancer biology.

## The challenges

A normal diploid cell contains about 6 pg of DNA, but present-day sequencing technologies require hundreds of nanograms of input material. To reach 500 ng of DNA, the genomes of about 80,000 cells are needed, or about 16 rounds of amplification from a single-cell genome. Various whole-genome amplification (WGA) methods have been developed and commercialized, each with specific advantages over the other, but no one method has dominated for all purposes [4]. Multiple displacement amplification (MDA) is a preferred method in the field for attaining broad genome coverage and SNV detection. However, all current WGA methods invariably cause artefacts: some loci or alleles are amplified more than others and some may not amplify at all. Single-base errors are made by the DNA polymerase despite its proof-reading ability, and DNA chimeras are fabricated. Given that differences between closely related cells can be very minor - a normal cell division introduces roughly one single-base change in a 6 Gb diploid genome - the errors produced even by polymerases with low error rates, such as the phi29 polymerase used in MDA, will quickly overwhelm the real differences. A recent study estimated the per-cycle per-base error rate for MDA to be about 3.2 × 10^−6^ [4], which would lead to about 19,000 false variants with each round of amplification of a human diploid genome.

To somewhat temper the errors, Navin and colleagues [1] first sorted and lysed single nuclei in a PCR tube, then performed a time-limited MDA reaction and subsequently selected, using a 22-chromosome quantitative PCR panel, cells without locus drop out in their amplification product, before conventional exome library preparation and sequencing.

An important quality metric for SNV and indel detection following single-cell WGA sequencing is the breadth of genome coverage (or in this case exome coverage), defined as the percentage of nucleotides of the genome (or exome) covered by at least one read. The authors [1] demonstrate that SNES can capture 90% of the exome in G0/1-phase cells and 96% in G2/M-phase cells - that is, in cells before and after DNA replication, respectively. This confirms that MDA can take a broad snapshot of the genomic landscape of a cell, and additionally indicates that having more DNA at the start of amplification has a positive effect. However, to call SNVs and indels at a particular location, that locus of the reference genome has to be layered with multiple sequencing reads of the cell. Using SNES, 73% and 84% of the exome reached sufficient coverage depth for genetic variant calling. These numbers illustrate the current state-of-the-art in single-cell sequencing: similar numbers were reported for other workflows that used MDA before single-cell exome sequencing [5].

Other important metrics include false-discovery rates and allele drop-out rates. Quantification of the number of errors introduced following SNES revealed that 26 errors per Mb were introduced following WGA by MDA; these were mostly false G:C > A:T transitions, although false T:A > C:G transitions and C:G > A:T transversions were also introduced [1,5]. Although this illustrates that single-cell sequencing technologies still have a long way to go, various groups have now observed that the large majority of errors of WGA occur at random sites and very few are recurrently observed in multiple single cells. This means that, in principle, variants observed in two (but preferably more) cells can be reliably called genuine [1,5]. In contrast, allelic drop-outs of known heterozygous SNPs amounted to 31% and 21% for G1/0- and G2/M-phase nuclei, respectively, when only regions with sufficient depth of coverage in the single cell were considered. Importantly, in contrast to false-positive errors, allelic dropouts can occur at recurrent positions in multiple single cells [1], complicating the interpretation of loss-of-heterozygosity events.

## Single-cell genomics to dissect intra-tumor heterogeneity

The emergence and evolution of cancer is driven by somatic changes to the genome. To understand how cancers develop and evolve, which cells enter the circulation and metastasize, and ultimately how resistance to treatment develops, we need to identify and study the different cell populations that can occur within cancer, and investigate how these populations are related. Although we can infer a rough architecture of the tumor’s subclones from sequencing bulk DNA of the tumor [6], the ultimate resolution to study intra-tumor heterogeneity is at single-cell level. Sequencing thousands or millions of individual cancer cells of many different tumors would allow a truly holistic view of tumor evolution, if the artefacts and error rate can be mitigated. In addition, the costs of such large-scale experiments are still prohibitive, mainly because of the cost of sequencing itself, but also because of the cost of amplification and library construction. Navin and colleagues [1] report a total cost of approximately 30 US dollars per cell to generate single-cell libraries (excluding exome capture). However, with cost-effective sequencing platforms as the Illumina HiSeq X ten, whole-genome sequencing of human single cells may now gradually move to an affordable high-throughput scale that will allow unique insights into cancer biology, and it may soon outrun the use of single-cell exome sequencing. Nevertheless, exome sequencing and, in particular, cost-effective custom targeted resequencing protocols, for example for cancer gene panels, will remain important research tools for single-cell analyses.

One caveat of the present SNES method [1] in the context of cancer genomics is the potential biases introduced by the 22-chromosome quantitative PCR quality control step. Because cancer cells may acquire copy number alterations, this quality control will behave differently for different subclonal populations. Such potential biases need to be kept in mind when designing an experiment.

## Exploring the role of somatic variation in other diseases and normal development

Somatic mutations during development may be neutral, contribute to the spectrum of normal phenotypic variation, or cause disease - not only cancer, but potentially also various other diseases [7]. For instance, it is thought that Joseph Merrick, an Englishman who lived in the late 19th century and whose life story was featured in the play and movie ‘The Elephant Man’, had Proteus syndrome, which is caused by a somatic activating mutation in the *AKT1* gene, leading to overgrowth of skin, connective and other tissues. Not only such very rare diseases (less than one case per million), but also more common diseases, including a variety of neurological disorders, have been associated with somatic mutations [7]. As occasional DNA alterations accumulate with each cell cycle following conception, such new mutations are inherited by descending daughter cells, and cell lineages can thus be tracked, as was recently demonstrated for the human brain using single-cell sequencing [8]. Somatic mutations may be prevalent throughout our body, some confined to a single cell, others scattered across ectoderm-, mesoderm- and endoderm-derived tissues, the building blocks of our organs. Therefore, in studies of normal developmental and disease processes, we expect that single-cell sequencing will have a transformative role. Error rates and artefacts still plague such studies, although this study [1] represents one step along the way to reduce those.

## The future

Technologies in which sequencing reads can be traced to the molecule of origin in the DNA sample have demonstrated their usefulness for mitigating sequencing error and artefacts for bulk DNA samples [9]. Similar principles translated to single-cell or single-nucleus sequencing technology may be able to further improve the reliability of genetic variants called following innovation in both experimental and computational techniques. In addition, reducing reaction time of WGA reactions and their reaction volumes to nanoliter scales have demonstrated a positive effect on bias and artefacts, and will lead to further advances.

Finally, methods for DNA and RNA sequencing of the same single cell have emerged [10]. Such integrated analyses of the cell’s transcriptome and genome and eventually also its epigenome and proteome will enable a more thorough understanding of the extent, function and evolution of cellular heterogeneity in normal development and disease.

## Abbreviations

MDA: Multiple displacement amplification
SNES: Single-nucleus exome sequencing
SNP: Single-nucleotide polymorphism
SNV: Single-nucleotide variant
WGA: Whole-genome amplification

## References

[1] Leung ML, Wang Y, Waters J, Navin NE (2015). SNES: single nucleus exome sequencing. Genome Biol.

[2] Van Loo P, Voet T (2014). Single cell analysis of cancer genomes. Curr Opin Genet Dev.

[3] Navin N, Kendall J, Troge J, Andrews P, Rodgers L, McIndoo J (2011). Tumour evolution inferred by single-cell sequencing. Nature.

[4] de Bourcy CF, De Vlaminck I, Kanbar JN, Wang J, Gawad C, Quake SR (2014). A quantitative comparison of single-cell whole genome amplification methods. PLoS One.

[5] Hou Y (2012). Single-cell exome sequencing and monoclonal evolution of a JAK2-negative myeloproliferative neoplasm. Cell.

[6] Nik-Zainal S, Van Loo P, Wedge DC, Alexandrov LB, Greenman CD, Lau KW (2012). The life history of 21 breast cancers. Cell.

[7] Biesecker LG, Spinner NB (2013). A genomic view of mosaicism and human disease. Nat Rev Genet.

[8] Evrony GD, Lee E, Mehta BK, Benjamini Y, Johnson RM, Cai X (2015). Cell lineage analysis in human brain using endogenous retroelements. Neuron.

[9] Schmitt MW, Kennedy SR, Salk JJ, Fox EJ, Hiatt JB, Loeb LA (2012). Detection of ultra-rare mutations by next-generation sequencing. Proc Natl Acad Sci U S A.

[10] Dey SS, Kester L, Spanjaard B, Bienko M, van Oudenaarden A (2015). Integrated genome and transcriptome sequencing of the same cell. Nat Biotechnol.

